# Quantitative Analysis of Flavonoids in *Glycyrrhiza uralensis* Fisch by ^1^H-qNMR

**DOI:** 10.1155/2021/6655572

**Published:** 2021-01-18

**Authors:** Ping Yu, Qian Li, Yanmei Feng, Yuying Chen, Sinan Ma, Xiaoqin Ding

**Affiliations:** Gansu Provincial Key Laboratory of Aridland Crop Science, College of Agronomy, Gansu Agricultural University, Lanzhou 730070, China

## Abstract

**Objective:**

To establish a method for simultaneous determination of liquiritin, liquiritigenin, and isoliquiritinin glycyrrhizin using hydrogen nuclear magnetic resonance quantitative technology (^1^H-qNMR). *Methodology*. Deuterated dimethyl sulfoxide was used as the solvent, and dichloromethane was used as the internal standard. The probe temperature was 298.0 K, the pulse sequence was Zg30, the number of scans was 16, and relaxation delay (*D*1) was 10 s. Quantitative characteristic signal peaks were *δ* 4.891∼4.878 ppm, *δ* 8.187∼8.172 ppm, and *δ* 6.790∼6.776 ppm for liquiritin, isoliquiritin, and liquiritigenin, respectively.

**Results:**

The experimental result showed that the content of flavonoids in Licorice, from Chifeng, Inner Mongolia, was the highest.

**Conclusion:**

In this study, a new method for determination of three flavonoids in Licorice using ^1^H-qNMR was established. This experimental method has the advantages of accuracy, efficiency, and economy. It lays a foundation for the study on the determination of flavonoids content in licorice by proton nuclear magnetic resonance spectroscopy.

## 1. Introduction

*Glycyrrhiza uralensis* Fisch. is a popular Chinese herbal medicine derived from the dried roots and rhizomes of *Glycyrrhiza uralensis* Fisch. species (Leguminosae family) [1]. It is one of the oldest and most popular herbal medicines in the world and was recorded in many Asian and European pharmacopoeias including China. In China, *Glycyrrhiza uralensis* Fisch. is called Gan-Cao, which means “sweet weed”. As a tonifying herbal medicine, Gan-Cao is extensively used in the traditional Chinese medicine (TCM) and appears as a component herb in about 60% of all TCM prescriptions [2]. In addition to medicinal usage, Gan-Cao is also used as a cake additive in food; its sweetness is one-hundred times that of sucrose. It also has applications in cosmetics, tobacco, and animal husbandry, etc. Gan-Cao shows a variety of pharmacological activities, including antiulceric, antiinflammatory, antispasmodic, antioxidative, antiallergic, antiviral, antidiabetic, anticancer, antidepressive, hepatoprotective, expectorant, and memory enhancing activities [3–5]. It is mainly used in clinical treatment of inflammation, cardiovascular, and cerebrovascular diseases, oxidative aging, tumors, etc [6–9]. The main active ingredients of *Glycyrrhiza uralensis* Fisch. include *Glycyrrhiza uralensis* Fisch. saponin and *Glycyrrhiza uralensis* Fisch. Flavonoids [10] and the liquiritin, liquiritigenin, and isoliquiritin are the main components of flavonoids. Although *Glycyrrhiza uralensis* Fisch. is considered as Generally Recognized as Safe (GRAS) for use in food by FDA (21 CFR 184.1408), large amounts may result in severe hypertension, hypokalemia, and other signs of mineralocorticoid excess [11, 12]. Therefore, quality control is critical to ensure the efficacy and safety of *Glycyrrhiza uralensis* Fisch.

Therefore, the content of liquiritin, liquiritigenin, and isoliquiritin in *Glycyrrhiza uralensis* Fisch. was determined in this study. Analytical methods of *Glycyrrhiza uralensis* Fisch. flavonoids include high-performance liquid chromatography (HPLC), thin-layer chromatography (TLC), and gas chromatography (GC). Some people also used gravimetric methods to measure the content of *Glycyrrhiza uralensis* Fisch. Flavonoids [13]. HPLC and GC are the most commonly used methods for quality control of TCM [14, 15]. However, their detectors are dependent on differing physical properties of the analytes. Thus, quantitative analysis of *Glycyrrhiza uralensis* Fisch. using these traditional methods requires two or more instruments. Considering the high requirement for efficacy and safety of *Glycyrrhiza uralensis* Fisch., an easier and faster method is needed for routine quality control [16].

Nuclear magnetic resonance (NMR) spectroscopy is commonly used for structure elucidation, but the potential for its quantitative ability is increasing nowadays. The ^1^H-qNMR method could simultaneously detect multiple components in very short time (1–5 mins) using a very cheap internal standard reference, which has found applications in quantitative analysis of natural products and other areas [12, 17–34]. In our previous study, we have developed a quantitative NMR (qNMR) method for *Angelica dahurica* and *Angelicae Pubescentis* Radix analysis [35, 36]. Major peaks in ^1^H-NMR and MS spectra contributing to the discrimination among species were assigned as those of glycyrrhizin, 4-hydroxyphenyl acetic acid, and glycosidic conjugates of liquiritigenin/isoliquiritigenin.

**Figure 1:**
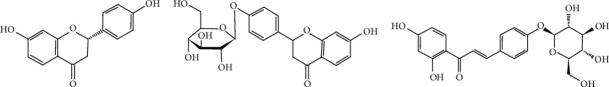
Structures of the reference standard (flavonoids): (a) liquiritigenin, (b) liquiritin, and (c) isoliquiritin.

In previous studies, Farag et al. reported the metabolite profiling and fingerprinting of medicinal licorice roots [37], and the ^1^H-NMR method was used to qualitatively identify the unknown components of the established fingerprints. *Glycyrrhiza uralensis* Fisch was investigated though centrifugal partition chromatography by Simmler et al. [38], and qNMR was used to determine the purity and residual complexity of the isolated compounds through orthogonal analysis. As known to us, there have been no reports about the simultaneous determination of liquiritin, liquiritigenin, and isoliquiritin by qNMR method, which are the active constituents of *Glycyrrhiza uralensis* Fisch. Therefore, the purpose of this study was firstly to simultaneously quantify the three main components liquiritin, liquiritigenin, and isoliquiritin. in *Glycyrrhiza uralensis* Fisch. by ^1^H-qNMR. The structural formula of the three components is shown in Figure 1.

## 2. Materials and Methods

### 2.1. Materials and Reagents

The reference standard of liquiritin (≥98%), liquiritigenin (≥98%), and isoliquiritin (≥98%) was purchased from Chengdu coming DE biological technology co., LTD (Sichuan, China). The internal standard dichloromethane (≥99%) was purchased from Sinopharm Group Chemical Reagent Co., Ltd. (Shanghai, China). DMSO-d_6_ (>99.8%) was purchased from Cambridge Isotope Laboratories, Inc. Other chemicals used in this work were of analytical grade.

The plant materials of *Glycyrrhiza uralensis* Fisch. were collected from Chifeng (Inner Mongolia, China), Hangqi (Inner Mongolia, China), Baotou (Inner Mongolia, China), Yanchi (Ningxia, China), Longxi (Gansu, China), and Taklamakan Desert (Xinjiang, China) and were identified as a piece of dried root and rhizome of the legume *Glycyrrhiza uralensis* by Professor Chen Yuan from the Department of Chinese Herbal Medicine of Gansu Agricultural University. The plant material samples were smashed with a pulverizer and passed through a 40-mesh sieve. The dried root sample used to determine repeatability, stability, and recovery was collected from Chifeng (Inner Mongolia, China).

### 2.2. Instrumentation

The nuclear magnetic resonance spectrometer (Bruker Advance III 600); AL-104 electronic analytical balance (Cixi Tiandong Weighing Apparatus Factory); KQ-500B ultrasonic cleaning instrument (Shenzhen Dekang Technology Co., Ltd.); rotary evaporator R-101N (Zhengzhou Great Wall Science and Industry Co., Ltd.); and constant temperature water bath (Jiangsu Zhengji Co., Ltd.) were used.

### 2.3. NMR Analytical Method Development

**Figure 2:**
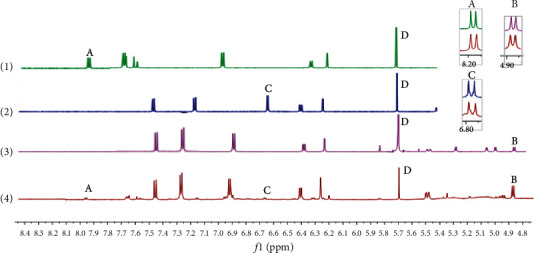
^1^H-NMR spectra of isoliquiritin (1), liquiritigenin (2), liquiritin (3), and *Glycyrrhiza uralensis* Fisch. Extract (4), obtained at 600 MHz in DMSO-d_6_ solvent. The quantitative signals of isoliquiritin (A), liquiritin (B), liquiritigenin (C), and dichloromethane (D) were at *δ* 8.187–8.172 ppm, *δ* 4.891–4.878 ppm, *δ* 6.83–6.85 ppm, and *δ* 5.75 ppm, respectively.

1. Preparation of inner standard solution: 100 *μ*L dichloromethane was dissolved in 5 mL DMSO-d_6_, and an internal standard solution of 26.5 mg/mL was prepared.
2. Preparation of samples for ^1^H-qNMR analysis: we have adopted the Chinese Pharmacopoeia Commission extraction method [38] for sample preparation. Decoction pieces of *Glycyrrhiza uralensis* Fisch. were crushed in a pulverizer and passed through a 40-mesh sieve. 10 g of *Glycyrrhiza uralensis* Fisch. powder was precisely weighed and put it in ethyl acetate and back flow for 1 h (material-liquid ratio 1 : 20 g/mL). Then, the filter residue was taken and put it in methanol back flow for 1 h (material-liquid ratio 1 : 15 g/mL). The filtrate was made into an extract and dissolved in water. The solution was washed three times with n-butyl alcohol, and the filtrates were combined to make extract.
3. Preparation of standard solution: we followed the methods of [22], 5 mg of isoliquiritin, liquiritigenin, and liquiritin were accurately weighed and put in DMSO-d_6_ (480 *μ*L) and 20 *μ*L dichloromethane solution (internal standard reference) were added, respectively. The standard solutions of isoliquiritin, liquiritigenin, and liquiritin were obtained, respectively. 30 mg of extract was accurately weighed and dissolved in 480 *μ*L DMSO-d_6_, and 20 *μ*L methylene chloride solution was added. The NMR spectra of extract and isoliquiritin, liquiritigenin, and liquiritin are shown in Figure 2.

### 2.4. ^1^H-NMR Spectroscopy

In this work, a 600 MHz NMR spectrometer was used to obtain the ^1^H-NMR spectra data, and the data were analyzed with MestReNova software. Using the experimental model of [26] to optimize the acquisition conditions and follow the contents: the spectral width: 11904 Hz, acquisition time: 3.78 s, *D*1 : 10 s, the pulse sequence was Zg30, the number of scans was 16, and the probe temperature was 298.0 K.

### 2.5. Methodology Validation

In this work, the following parameters were used to validate the developed method: linearity, limit of detection, limit of quantitation, precision, repeatability, stability, and recovery.

The linearity of liquiritin, isoliquiritin, and liquiritigenin is shown in Table 1, which indicated that the constructed analytical curves presented a satisfactory linearity.

The limit of detection and quantitation can be determined by the methods of [22]. LOD = 3.3 *σ*/s and LOQ = 10 *σ*/s. The detection and quantitation limits of liquiritin were 0.023 mg/mL and 0.070 mg/mL, the detection and quantitation limits of liquiritigenin were 0.022 mg/mL and 0.068 mg/mL, and the detection and quantitation limits of isoliquiritin were 0.003 mg/mL and 0.010 mg/mL, respectively.

The same reference standard was measured six times to get precision results; six sample solutions were used to get repeatability results, and the same sample was measured within 12 h to get the stability results. The standard deviations of precision for liquiritin, isoliquiritin, and liquiritigenin were 1.18%, 1.13%, and 1.24%, respectively. The standard deviations of repeatability for liquiritin, isoliquiritin, and liquiritigenin were 1.68%, 2.29%, and 4.43%, respectively. The standard deviations of stability for liquiritin, isoliquiritin, and liquiritigenin were 1.08%, 2.25%, and 1.24%, respectively.

Three equal amounts of licorice extracts were accurately weighed, and the contents of liquiritin, liquiritigenin, and isoliquiritinin were calculated. Then, the double amount of standard solution was added to the licorice extracts solution, and the recovery results are shown in Table 2.

## 3. Results and Discussion

### 3.1. Selection of Solvent and Internal Standard

The results showed that the *Glycyrrhiza uralensis* Fisch. extract had good solubility in DMSO-d_6_ solvent through the preliminary experiment. Besides, the target analytes were stable and easy to separate in the solvent. Therefore, DMSO-d_6_ was selected as the solvent for qNMR analysis.

As a qNMR experiment, the internal standard should have high stability, high purity, easy weighing nature, and no effect on the peaks of the materials to be tested. According to the previous work experience, cresol, pyrazine, maleic acid, and dichloromethane were selected for experiments. The results showed that only dichloromethane had good stability during the experiment and was able to overcome peak-to-peak mutual influence. Therefore, dichloromethane was selected as the internal standard.

### 3.2. Choice of Relaxation Delays

The instrument parameters such as D1 have a greater impact on the accuracy of the test results. Studies have shown that D1 must be set long enough (>5T1) in qNMR experiments to fully relax the nucleus and then be integrated. The impact of D1 on the experimental results was carried out. For the same sample to be tested, using the ratio of the quantitative peak and the internal standard peak (Au/As) as a reference, the D1 values for liquiritin, liquiritigenin, and isoliquiritin were 5 s, 10 s, 20 s, 30 s, and 40 s. When the D1 value is greater than 10 s, the Au/As value will not change anymore. Hence, 10 s was select as relaxation delays to save test time.

### 3.3. Quantitative Results

**Figure 3:**
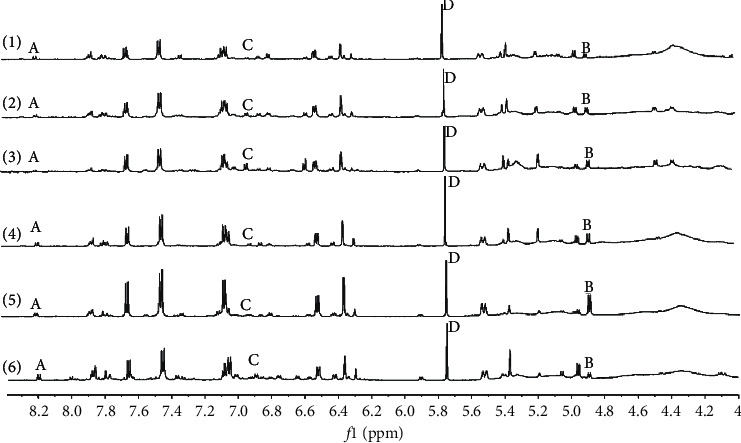
^1^H-NMR spectra of *Glycyrrhiza uralensis* Fisch. from different regions: Hangqi (1); Yanchi (2); Longxi (3); Baotou (4); Chifeng (5); Taklamakan Desert (6). The signals of four contents were at *δ* 8.187 − *δ* 8.172 ppm for isoliquiritin (A), *δ* 4.891-*δ*4.878 ppm for liquiritin. (B), *δ* 6.790-*δ* 6.776 ppm for liquiritigenin (C), and *δ* 5.75 ppm for dichloromethane (D).

The contents of three flavonoids in *Glycyrrhiza uralensis* Fisch. were determined by the ^1^H-qNMR for the first time, and the results are shown in Table 3 and Figure 3.

The experimental result showed that *Glycyrrhiza uralensis* Fisch. from Chifeng, Inner Mongolia, had the highest content of flavonoids.

The HPLC method was used to verify the ^1^H-qNMR. The above extract was accurately weighed to 10.00 mg and completely dissolved in 10 mL of 70% absolute ethanol by ultrasonic treatment which was transferred to a sample vial for HPLC determination. Each batch of plant material was performed in triplicate. The ^1^H-qNMR method was also used to determine the composition of three batches of plant materials, each batch of samples was repeated 3 times, and the results were compared with HPLC. The details of the HPLC are shown in Supplementary Materials. The results are shown in Table 4. There was no significant difference between the two measurement methods.

## 4. Conclusion

In this study, a ^1^H-qNMR method was developed for the simultaneous determination of liquiritin, isoliquiritin, and liquiritigeninin in *Glycyrrhiza uralensis* Fisch. for the first time. This work provided a new effective method for quality control of *Glycyrrhiza uralensis* Fisch.

## Figures and Tables

**Table 1:** Standard curves of three flavonoids in *Glycyrrhiza uralensis* Fisch.

Compound	Regression equation	*R* ^2^	Linearity (mg/ml)
Liquiritin	*y* = 3.9428*x* + 0.0289	0.9996	0.25∼2
Isoliquiritin	*y* = 8.6091*x* − 0.0137	0.9999	0.3∼2
Liquiritigenin	*y* = 2.3963*x* − 0.0132	0.9997	0.3∼2

**Table 2:** Recovery (%) of the flavonoids in *Glycyrrhiza uralensis* Fisch. by the ^1^H-qNMR method.

	Liquiritin		Isoliquiritin		Liquiritigenin	
	Recovery	RSD	Recovery	RSD	Recovery (%)	RSD (%)
1	106.35	1.16	101.09	1.66	107.32	1.86
2	108.80		100.00		107.38	
3	108.00		103.30		110.83	

**Table 3:** Content (%) of three flavonoids in *Glycyrrhiza uralensis* Fisch. by the ^1^H-qNMR method.

Sample	Liquiritin (%)	Liquiritigenin (%)	Isoliquiritin (%)
Sample A	0.24	0.11	0.18
Sample B	0.50	0.18	0.30
Sample C	0.17	0.05	0.01
Sample D	0.38	0.07	0.09
Sample E	1.92	0.22	0.29
Sample F	0.29	0.14	0.13

*Note*. Sample A (Hangqi), Sample B (Yanchi), Sample C (Longxi), Sample D (Baotou), Sample E (Chifeng), and Sample F (Taklamakan Desert).

**Table 4:** Comparison of contents of three components between ^1^H-qNMR and HPLC.

	Liquiritin		Isoliquiritin		Liquiritigenin	
	HPLC	NMR	HPLC	NMR	HPLC (%)	NMR (%)
1	16.32	16.68	4.63	4.59	0.87	0.94
2	16.30	16.65	4.66	4.60	0.88	0.93
3	16.32	16.66	4.64	4.60	0.90	0.94

## Data Availability

The original ^1^H-NMR spectral data and the analysis method of the data used to support the findings of this study are available from the corresponding authors upon request.

## Supplementary Materials

Supplementary Materials Supplementary Materials are about “Sample Analysis and Verification by HPLC”. Figure 1: HPLC chromatograms of medicinal material extract (A) and mixed reference solution (B). Table 1: regression data for three bioactive compounds obtained by HPLC results. Table 2: the precision, repeatability, and stability of three flavonoids. Table 3: the recovery of three flavonoids. Table 4: comparison of H-qNMR and HPLC.
