# Supplementary material for: Quantitative Analysis of Flavonoids in Glycyrrhiza uralensis Fisch by 1H-qNMR
Source: J Anal Methods Chem. 2021 Jan 18;2021:6655572. doi: 10.1155/2021/6655572 (PMC7834775; doi:10.1155/2021/6655572)
Supplement: Supplementary Materials — are about “Sample Analysis and Verification by HPLC”. Figure 1: HPLC chromatograms of medicinal material extract (A) and mixed reference solution (B). Table 1: regression data for three bioactive compounds obtained by HPLC results. Table 2: the precision, repeatability, and stability of three flavonoids. Table 3: the recovery of three flavonoids. Table 4: comparison of H-qNMR and HPLC. [file 6655572.f1.docx]

**Supporting information**

**Quantitative analysis of flavonoids in** **Glycyrrhiza uralensis Fisch by ^1^H-qNMR**

Ping Yu^#^, Qian Li^#^*, Yanmei Feng, Yuying Chen, Xiaoqin Ding, Sinan Ma

(Gansu Provincial Key Laboratory of Aridland Crop Science, College of Agronomy, Gansu Agricultural University, Lanzhou 730070, China)

^*^Corresponding author: Qian Li, Email: [liqian1984@gsau.edu.cn](mailto:liqian1984@gsau.edu.cn)

^#^These authors contributed equally.

**Sample Analysis and Verification by HPLC**

1. **HPLC conditions and the preparation of mixed standard solution**

The chromatographic column was Symmetry® C18(4.6 mm × 250 mm,5 μm); the mobile phase was acetonitrile-0.005% phosphoric acid aqueous solution. The gradient elution: 0～20 min, 0%～12% acetonitrile, 20～45 min, 12%～32% acetonitrile; 45～75 min, 32%～70% acetonitrile; 75～76 min, 70%～98% acetonitrile ;76～80 min, 97%～12% acetonitrile; and the detection wavelength was set at 254 nm. The column was operated at 25 ℃ with the mobile phase at a constant flow rate of 1 mL/ min.

Firstly, the appropriate amounts of standard substances were accurately weighed and placed in 10 mL volumetric flasks with 70% ethanol. (the mass concentration of liquiritin, isoliquiritin, liquiritigenin were 0.45, 0.26, 0.35 mg/mL);


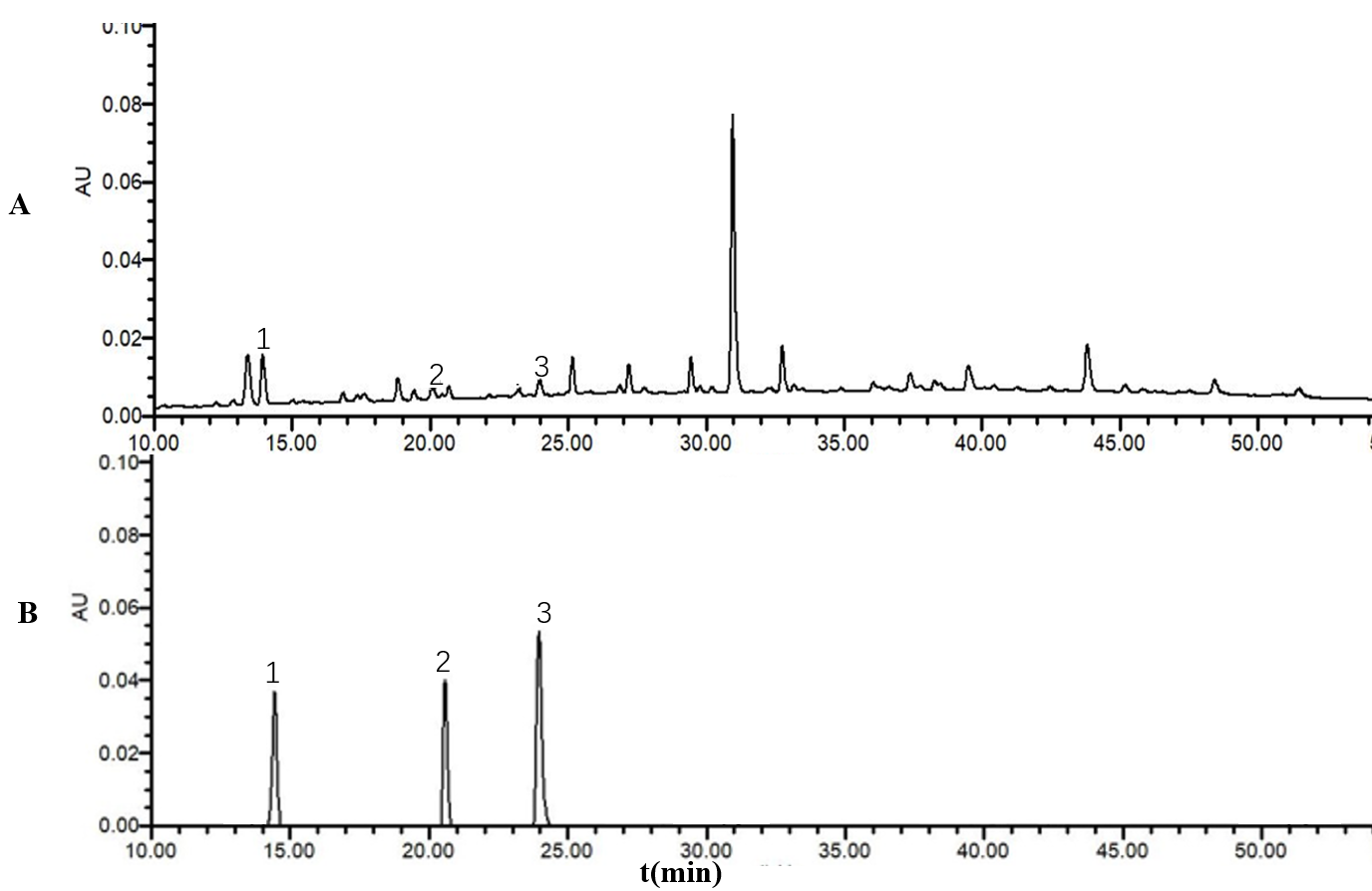


Figure 1 HPLC chromatograms of medicinal material extract (A) and mixed reference solution (B). Note: 1 is liquiritin, 2 is isoliquiritin, 3 is liquiritigenin

1. **Linearity**

According to the HPLC results of five different concentrations of reference substance, the function relationship between concentration (X) and peak area (Y) was obtained, as shown in Table 1, The R^2^ of liquiritin, liquiritigenin, isoliquiritin were both greater than 0.999, indicating a good linear relationship.

Table 1 Regression data for three bioactive compounds obtained by HPLC results.

| Analyte | Calibration curvea | Linearity range (μg/μL) | ***R^2^*** | LOD | LOQ |
| --- | --- | --- | --- | --- | --- |
| Liquiritin | y=6000000x-3761.8 | 0.018~0.09 | 0.9997 | 0.0020492 | 0.0062096 |
| Isoliquiritin | y=9000000x-1581.9 | 0.0104~0.052 | 1 | 0.0002908 | 0.0008812 |
| Liquiritigenin | y=10000000x-5257.6 | 0.014~0.07 | 1 | 0.0004212 | 0.0012762 |

1. **Precision, repeatability, stability and recovery**

The standard mixed solution of Glycyrrhiza uralensis was taken and injected 6 times continuously under the same chromatographic conditions. The peak area values were recorded and the RSD were shown in the Table 2.

6 samples of the solution obtained were precisely injected to the HPLC system, the samples according to the chromatographic conditions. The RSD of liquiritin, isoliquiritin, liquiritigenin were showed on the Table 2.

10 μL of the test solution obtained was analyzed under the chromatographic conditions at 1, 2, 8, 16, 24 h. The result showed that the RSD of the peak area of liquiritin, isoliquiritin, liquiritigenin were shown in the Table 2.

The recovery rate of standard addition = (measured value of spiked sample-measured value of sample) ÷ spiked amount × 100%. Thus, the sample solution with a known content The sample solution with a known content is repeated three times for content determination. The results were shown in Table 3.

Table 2 The Precision, repeatability, stability of three flavonoids

| Analyte | Precision | stability | repeatability |
| --- | --- | --- | --- |
| Liquiritin | 0.33% | 1.93% | 1.43% |
| Isoliquiritin | 1.05% | 1.77% | 2.01% |
| Liquiritigenin | 0.45% | 1.32% | 1.77% |

Table 3 The recovery of three flavonoids

| Analyte | Times | recovery | RSD |
| --- | --- | --- | --- |
| Liquiritin | 1 | 104.17% | 0.03% |
|  | 2 | 104.22% |  |
|  | 3 | 104.22% |  |
| Isoliquiritin | 1 | 101.57% | 0.58% |
|  | 2 | 100.72% |  |
|  | 3 | 101.84% |  |
| Liquiritigenin | 1 | 104.09% | 0.57% |
|  | 2 | 103.09% |  |
|  | 3 | 103.03% |  |

1. **Sample analysis**

The above extract was accurately weighed to 10.00 mg and completely dissolved in 10 mL of 70% absolute ethanol by ultrasonic treatment which was transferred to a sample vial for HPLC determination. Each batch of plant material was performed in triplicate.

The H-qNMR method was also used to determine the composition of three batches of plant materials, each batch of samples was repeated 3 times, and the results were compared with HPLC. The results were shown in Table 4. There was no significant difference between the two measurement methods.

Table 4 Comparison of H-qNMR and HPLC

|  | Liquiritin | | Isoliquiritin | | Liquiritigenin | |
| --- | --- | --- | --- | --- | --- | --- |
|  | HPLC | NMR | HPLC | NMR | HPLC | NMR |
| 1 | 16.32% | 16.68% | 4.63% | 4.59% | 0.87% | 0.94% |
| 2 | 16.30% | 16.65% | 4.66% | 4.60% | 0.88% | 0.93% |
| 3 | 16.32% | 16.66% | 4.64% | 4.60% | 0.90% | 0.94% |
